# Host Range and Impact of *Dichrorampha aeratana*, the First Potential Biological Control Agent for *Leucanthemum vulgare* in North America and Australia

**DOI:** 10.3390/insects12050438

**Published:** 2021-05-12

**Authors:** Sonja Stutz, Rosemarie De Clerck-Floate, Hariet L. Hinz, Alec McClay, Andrew J. McConnachie, Urs Schaffner

**Affiliations:** 1 CABI, Rue des Grillons 1, CH-2800 Delémont, Switzerland; h.hinz@cabi.org (H.L.H.); u.schaffner@cabi.org (U.S.); 2 Agriculture and Agri-Food Canada, Lethbridge Research and Development Centre, 5403—1 Ave. S., Lethbridge, AB T1J 4B1, Canada; rosemarie.declerck-floate@canada.ca; 3 12 Roseglen Private, Ottawa, ON K1H 1B6, Canada; alec.mcclay@shaw.ca; 4 Weed Research Unit, New South Wales Department of Primary Industries, Biosecurity and Food Safety, Orange, NSW 2800, Australia; andrew.mcconnachie@dpi.nsw.gov.au

**Keywords:** host-specificity testing, impact studies, ox-eye daisy, weed biological control, Asteraceae, Tortricidae

## Abstract

**Simple Summary:**

Oxeye daisy, a Eurasian member of the daisy family, has become invasive in several parts of the world, including North America and Australia. We investigated whether a root-feeding moth found closely associated with oxeye daisy in Europe could be used as a biological control agent for the plant when weedy. We found that the moth could develop on 11 out of 74 plant species that we tested in laboratory conditions when it was given no choice of plants. When the moths were given a choice of food plants outdoors, we found its larvae only on the ornamentals Shasta daisy and creeping daisy. Larval feeding had no impact on the weight and number of flowers of Shasta daisy, but larval feeding and plant competition reduced both measurements for oxeye daisy. We conclude that it is safe to release the moth species because it will not affect the ornamental value of Shasta daisy or creeping daisy and because it is unlikely to harm any other economically important or native species. Based on the moth’s preference for oxeye daisy, and that we expect it to contribute to the suppression of the weed, we propose its field release in North America and Australia.

**Abstract:**

We evaluated the potential of the European root-feeding moth *Dichrorampha aeratana* as a biological control agent for the invasive weed *Leucanthemum vulgare* (oxeye daisy) in North America and Australia. The taxonomic proximity of the ornamental Shasta daisy (*Leucanthemum × superbum*) to *L. vulgare* and its popularity in North America made finding sufficiently host-specific biological control agents a challenge. No-choice tests conducted with 74 non-target species revealed partial or complete larval development on 11 species. In multiple-choice oviposition and larval development tests that were conducted in field cages, larvae were found on five of these, however in multiple-choice tests conducted under open-field conditions, larvae were only found on the ornamentals Shasta daisy and creeping daisy (*Mauranthemum paludosum*). Larval feeding by *D. aeratana* had no measurable impact on Shasta daisy, but larval feeding and plant competition reduced the biomass and number of flower heads of *L. vulgare*. We conclude that *D. aeratana* is a suitable biological control agent because it will not affect the ornamental value of Shasta or creeping daisies and because it is unlikely to feed on any other economically important or native species. We also expect *D. aeratana* to contribute to the suppression of *L. vulgare* populations.

## 1. Introduction

*Leucanthemum vulgare* (Vaill.) Lam. (Asteraceae) (oxeye daisy) is a perennial forb native to Europe and western Asia. It has been introduced across the world as an ornamental or a seed contaminant and can now be found on all continents except Antarctica [1]. In North America, *L. vulgare* is common in the north-eastern and north-western states of the USA and in the eastern and western provinces of Canada [2]. In Australia, it is widespread in Victoria and Tasmania and becoming more abundant in New South Wales [3]. *Leucanthemum vulgare* is also known as an invasive weed in the Kashmir Valley [4] and its invasion risk is expected to increase with climate change due to an increase in suitable habitats [5]. *Leucanthemum vulgare* can form dense, extensive populations in pastures, meadows, forest openings, and along roadsides. It is generally avoided by grazing cattle, which in turn facilitates the development of large *L. vulgare* infestations that contribute to forage production losses in parts of western North America [2,6]. *Leucanthemum vulgare* has also become a problematic weed in conservation areas, such as the Kosciuszko National Park in New South Wales, where it is currently a threat to several rare species [3,7]. Application of herbicides to suppress *L. vulgare*, and/or of fertiliser to stimulate the growth of competing vegetation, are currently among the most effective methods to control *L. vulgare* temporally and/or locally, but there is a lack of methods suitable for the sustainable and long-term management of this weed across invaded landscapes [2,3,6]. In 2008 and 2015, a biological control programme against *L. vulgare* was therefore initiated in North America and Australia, respectively [7,8].

In Eurasia, the genus *Leucanthemum* is represented by 42 species [9], but none of these are native to North America or Australia. Aside from the diploid *L. vulgare*, the morphologically similar, but tetraploid *L. ircutianum* (Turcz.) Turcz. ex DC. has also been introduced to North America and potentially also to other parts of the world, but it is far less abundant than *L. vulgare* [10,11,12]. The genus *Leucanthemum* is phylogenetically relatively well isolated from species native to North America or Australia [13,14], which should facilitate the finding of sufficiently specific biological control agents for *L. vulgare*. However, the popularity of the closely related horticultural plant Shasta daisy *Leucanthemum* × *superbum* (Bergmans ex J. Ingram) Kent (sometimes also referred to as *L. maximum* (Ramond) DC.) in North America makes this more challenging [15]. Shasta daisy is a highly polyploid hybrid of *L*. *vulgare*, *L*. *maximum*, *L. lacustre* (Brot.) Samp., and another Asteraceae species that was developed by the famous American plant breeder, Luther Burbank in 1901 [16]. In North America, numerous cultivars of Shasta daisy are widely grown as ornamental plants, mostly in home gardens [17,18]. In Australia, Shasta daisy is a less popular garden or container grown ornamental, and is occasionally used in the cut flower industry.

Based on literature surveys, eight insect species were prioritised as potential biological control agents because records suggested their host ranges are restricted to *Leucanthemum* species; the root-mining tortricid moths *Dichrorampha aeratana* Pierce and Metcalfe and *D. baixerasana* Trematerra, the shoot-mining moth *D*. *consortana* Stephens, the root-feeding weevils *Cyphocleonus trisulcatus* Herbst and *Diplapion stolidum* (Germar), the root-galling tephritid fly *Oxyna nebulosa* (Wiedemann), the flower-head feeding tephritid fly *Tephritis neesii* (Meigen), and the flower-head feeding weevil *Microplontus campestris* (Gyllenhal). *Microplontus campestris*, *T. neesii*, and *D. stolidum* were subsequently dropped from the list of potential agents owing to a lack of impact on seed output or host specificity, while studies with most of the other species are still ongoing. Work on *C. trisulcatus* for North America was stopped as pre-release open-field studies showed that attack on Shasta daisy was similar as on *L. vulgare*, but this species is still considered as a potential biological control agent for Australia [15].

In this study, we evaluated the potential of *D. aeratana* as a biological control agent for *L. vulgare* in North America and Australia by experimentally assessing its host range and impact on target and non-target species. *Dichrorampha aeratana* is a univoltine species whose adults emerge in spring, typically from April to June. The females lay their eggs on the leaves and stems where the newly hatched larvae start mining down into the roots. During summer and autumn, the larvae feed internally and externally on the roots, rhizomes, or stem bases. Larvae develop through five instars, overwinter in the roots and pupate in early spring, typically in March. In the literature, *D. aeratana* is recorded exclusively from *L. vulgare* (presumably including *L. ircutianum*) [19,20]. In addition, larval survival of *D. aeratana* has been found to be negatively associated with an increasing ploidy level in the genus *Leucanthemum* [21], suggesting that the highly polyploid Shasta daisy may be a less suitable host plant for *D. aeratana* than the diploid *L. vulgare.* We recently discovered that *D. aeratana* has been reported from several locations in eastern North America, with the oldest records dating from 1992 [22,23]. However, the insect has not been recorded in western North America. The risk of *D. aeratana* using any non-target plants as hosts needs to be evaluated through host-range studies before permits can be obtained to release and distribute it as a biological control agent for *L. vulgare* in western North America.

To determine the fundamental host range of *D. aeratana*, i.e., the range of species on which the larvae are physiologically able to complete development [24], we carried out no-choice larval development tests. As a second and third step in the testing sequence, we conducted multiple-choice tests in cages and in the open field to estimate the ecological host range of *D. aeratana*, i.e., the range of species that are likely to be used as hosts if *D. aeratana* were released in North America or Australia [25,26,27]. In addition to the host-specificity tests, we also conducted an impact experiment to determine whether Shasta daisy, which was able to support the moth’s development under all test conditions, was negatively impacted by larval feeding by *D. aeratana.*

We also experimentally assessed the impact of *D. aeratana* on *L. vulgare*. Having a negative impact on the target weed is not only important for its successful control, but also because biological control agents reaching high population levels, without impacting the target weed, may lead to indirect non-target effects [28,29]. For several species, the impact of biological control agents has been shown to be enhanced if the target weed is exposed to other stress factors, such as competition from neighbouring plants [30]. *Poa pratensis* is a desired grass species frequently growing in pastures infested with *L. vulgare* in North America, and that can reduce the biomass and number of flower heads of *L. vulgare* when growing in competition [31]. We therefore assessed the separate and combined effects of larval feeding by *D. aeratana* and competition with Kentucky bluegrass *Poa pratensis* L. (Poaceae) on the growth and reproduction of the target weed *L. vulgare.*

## 2. Materials and Methods

### 2.1. Insect Rearing

The *D. aeratana* larvae and adults used in this study originated from a rearing colony established from larvae collected on *L. ircutianum* in Sonogno, Switzerland (46.349° N, 8.787° E, 910 m). *Dichrorampha aeratana* was continuously reared on potted plants of *L. vulgare* and *L. ircutianum* that were kept outdoors in gauze-covered field cages at CABI in Delémont, Switzerland (47.373° N, 7.326° E, 520 m) since 2011. To avoid inbreeding effects, the rearing colony was supplemented with field collected larvae almost every year since its initial establishment. In 2017 and 2018, respectively, eggs obtained from this rearing colony were imported to the quarantine facilities at the New South Wales Department of Primary Industries (NSW DPI) in Orange, Australia (permit no. 0001297526) and at Agriculture and Agri-Food Canada (AAFC) in Lethbridge, AB, Canada (permit no. P-2018-01867). Details on the rearing methods can be found in the Supplementary Materials File S1.

### 2.2. Test Plant Species

As part of the biological control project for North America, a test plant list was compiled using the centrifugal phylogenetic approach proposed by Wapshere [32] and revised by Briese [33] and Kelch and McClay [34]. This test plant list included: (i) Several populations of the target weed *L. vulgare* and of the closely related *L. ircutianum*, (ii) several cultivars of Shasta daisy (*L. × superbum*), (iii) North American native and economic plants in the family Asteraceae, especially in the tribe Anthemideae (based on the phylogeny published by Oberprieler et al. [14]), and (iv) plants with biochemical similarities with *L. vulgare*. The test plant list was submitted to the USDA-APHIS Technical Advisory Group for Biological Control Agents of Weeds and the Canadian Biological Control Review Committee in 2013, and additional species were added to the list based on comments provided by the reviewers and on our own reviews. This updated test plant list was subsequently reviewed by an Australian taxonomist and Asteraceae expert (Alexander Schmidt-Lebhun, CSIRO) and several species native to Australia, as well as additional horticultural species, were added. In total 74 non-target species were tested, of which 33 and 9 are native to North America and Australia respectively (Table 1). Plants from 24 *L. vulgare* and 8 *L. ircutianum* populations from North America, Europe, and Australia were used as controls (see File S1). The North American native *Aralia nudicaulis* L. (Araliaceae) and the Australian natives *Leptinella drummondii* (Benth.) D.G.Lloyd and C.J.Webb (Asteraceae) and *Cotula vulgaris* var. *australasica* J.H.Willis (Asteraceae) had been added to the test plant list, but were not available for testing. The majority of plants were grown from seeds two to four months before being used in tests to ensure that they would be large enough to be attractive for oviposition and to support larval development (see File S1 for details).

### 2.3. Host-Range Testing

All plant species were first tested in no-choice larval development tests and all except one species (*Cotula vulgaris* var. *vulgaris*) that supported larval development in these tests were subsequently exposed to females in multiple-choice tests in field cages and under open-field conditions. The multiple-choice tests evaluated the acceptance of these non-target species for oviposition when females were given a choice between *L. vulgare* and non-target species following the procedure suggested by Wapshere [35]. Since the presence of large numbers of Shasta daisy cultivars in multiple-choice tests may prevent *D. aeratana* females from ovipositing on other, less preferred hosts, separate multiple-choice tests were conducted to determine the acceptance of different Shasta daisy cultivars and the acceptance of more distantly related non-target species. Since in the native range adults of *D. aeratana* often emerge at the time its host species produce stems, and because in North America *L. vulgare* likely occur as rosettes and as plants with stems during *D. aeratana* adult emergence [2], we wanted to test whether *D. aeratana* has a preference for one of these two phenostages. We therefore included 3–4-month-old plants in the rosette stage as well as 15–16-month-old plants with stems in the multiple-choice tests.

Most of the no-choice tests and all of the multiple-choice tests were conducted at CABI in Delémont, Switzerland between 2011 and 2020. A few of the no-choice tests were conducted in quarantine at the New South Wales Department of Primary Industries (NSW DPI) in Orange, Australia and at Agriculture and Agri-Food Canada (AAFC) in Lethbridge from 2017–2021 (see—File S1 for details).

#### 2.3.1. No-Choice Larval Development Tests

No-choice larval development tests were carried out between 2011 and 2021. In spring, five larvae, no older than 24 h, were transferred onto the rhizomes, stem base, or leaf petioles of each potted plant. For each date a series of plants was set up, at least 20% of the plants were *L. vulgare* or *L. ircutianum*, as controls. After the transfer of larvae, most plants were placed in gauze-covered field cages and embedded in sawdust at CABI. At AAFC and NSW DPI, plants were placed under growth lights in the quarantine facilities maintained at 10–20 °C or 18–27 °C, respectively.

Most plants were dissected 4–6 months after they had been infested with larvae and the number of larvae found on each plant was recorded. To investigate whether *D. aeratana* could complete its development to the adult stage on non-target species in which larvae were found, a subset of control and non-target plants (each of which had been infested with 5 larvae in 2012, 2013, 2014, and 2018) were overwintered without being dissected in CABI’s garden. In addition, a subset of the larvae found on non-target and target plants in autumn 2012 were transferred onto new plants of the same plant species. In the following spring, these plants were individually covered with gauze bags and regularly checked for adult emergence.

#### 2.3.2. Oviposition and Development Test with Shasta Daisy in Field Cages

This test investigated whether: (i) Larval transfer tests gave similar results to oviposition tests, (ii) the proportion of eggs that developed through to adult differed between *L. vulgare*, *L. ircutianum*, and Shasta daisy, and (iii) the proportion of eggs that developed through to adult differed among Shasta daisy cultivars. For this, nine Shasta daisy cultivars as well as *L. vulgare* and *L. ircutianum* were exposed to females of *D. aeratana* in five gauze-covered field cages (2 m × 2 m × 1.6 m) in April 2014. Each field cage contained two plants of each Shasta daisy cultivar as well as of *L. vulgare* and *L. ircutianum*. Six egg-laying females were released into each of the cages. Two weeks later, all plants were searched for eggs and the number on each was recorded. Since the goal of this experiment was not to test for oviposition preferences, plants with fewer than two eggs (one *L. vulgare*, two *L. ircutianum*, and 32 Shasta daisy plants of all nine cultivars) were rearranged in two new field cages and another four females were released in each of these cages to ensure that all plants received eggs. The number of eggs was counted again two weeks later. In spring 2015, all plants were individually covered with gauze bags, and adult emergence was recorded from 21 April to 29 May.

#### 2.3.3. Multiple-Choice Oviposition and Larval Development Tests in Field Cages

Multiple-choice oviposition and larval development tests in field cages were conducted in 2012, 2013, 2016, 2017, and 2020. In each year, tests were conducted in 4–6 gauze-covered field cages (2 m × 2 m × 1.6 m) each containing three potted plants, each holding 3–4 non-target species or cultivar and *L. vulgare* and/or *L. ircutianum* as controls (see Table 2). The plants were randomly distributed within the field cages and the pots placed in sawdust. The plants did not touch each other or the net of the field cages. Between late April and early June, 6–12 egg-laying females were released in each of the field cages. In the 2012 test, all plants were inspected for eggs and the number counted (except for the *L. vulgare* plants with stems, where it was not possible to accurately count the eggs). The number of eggs was not recorded in subsequent years. In each year, all plants were dissected 3–5 months after the test had been set up. The number of larvae found during dissection was recorded and a subset of these from target and non-target plants were weighed and the maximum width of their head capsule measured to determine the larval instar.

#### 2.3.4. Multiple-Choice Oviposition and Larval Development Tests under Open-Field Conditions

**Figure 1:**
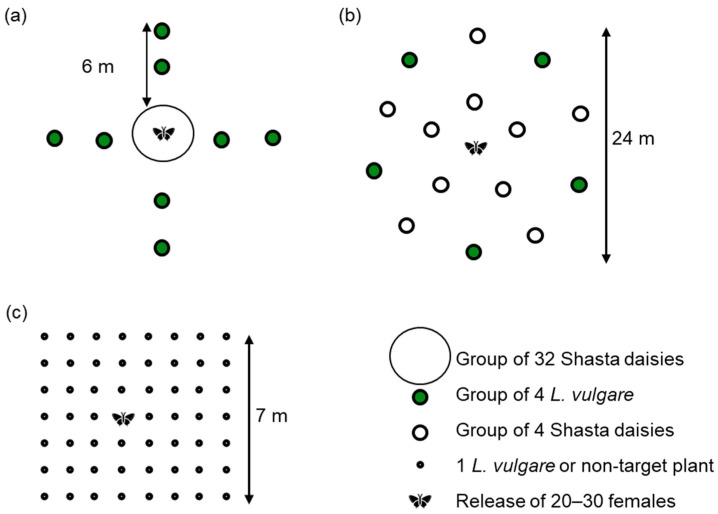
Designs of multiple-choice open-field tests conducted with *Dichrorampha aeratana* in (**a**) 2013, (**b**) 2014, and (**c**) 2014, 2017, and 2019.

Five multiple-choice open-field tests were established in a meadow at CABI in Delémont between 2013 and 2019. To better understand the host-selection behaviour of *D. aeratana*, we used three different test designs: The first design aimed to investigate whether females of *D. aeratana* released on a large patch of Shasta daisies would stay and oviposit on plants of this patch, or leave the patch to oviposit on smaller patches of *L. vulgare* further from the release point (Figure 1a). In 2013, we therefore set up a large patch of 36 potted plants consisting of three Shasta daisy cultivars (12 plants of each cultivar) surrounded by eight smaller patches, each consisting of two potted *L. vulgare* plants in the rosette stage and two potted *L. vulgare* plants with stems. The eight *L. vulgare* patches were set up at four different geographic orientations at a distance of 3 mand 6 m from the Shasta daisy patch. Since this test revealed that *D. aeratana* would oviposit on Shasta daisy plants situated close to the release point, we set up an additional open-field test in 2014, which aimed to test whether *D. aeratana* would also oviposit on Shasta daisy plants if they were exposed further away from the release point (Figure 1b). In this test, we exposed a total of 40 potted Shasta daisy plants from four cultivars in ten patches (each consisting of one plant of each cultivar) set up at 6 m and 12 m from the release point and 20 *L. vulgare* plants in five patches set up at 12 m from the release point. As the first two open-field test designs revealed that the distance and orientation from the release point did not influence the number of larvae found on plants (all *P* > 0.1), we used a simpler design for the remaining three open-field tests (Figure 1c). In these tests, 9–16 plants of 3–5 non-target and of *L. vulgare* were randomly arranged within a 7 m x 8 m plot with 1 metre of spacing between the plants. A total of 11 non-target species were exposed in this way in 2014, 2017, and 2019. In each of the five tests, potted plants were sunk in the soil to at least ¾ of the pot height and in April or May, 20 to 30 egg-laying females were released at the centre point of the study area. The plants were left in the meadow for 2–4 weeks and thereafter placed in gauze-covered field cages until they were dissected 3–5 months later. The number of larvae found during dissection was recorded and the weight and instar was recorded from a subset of the larvae found on target and non-target plants. As larvae of *D. aeratana* are not morphologically distinguishable from larvae of other *Dichrorampha* species, a few were identified by molecular analysis (see File S1) and the remaining larvae were placed on *L. vulgare* to be reared to adults for morphological identification. All larvae and emerged adults were identified as *D. aeratana*, and we therefore concluded that any *Dichrorampha* larvae found on *L. vulgare* (or any non-target plants) were very likely to be *D. aeratana*.

### 2.4. Impact of Dichrorampha aeratana on Shasta Daisy and Impact of D. aeratana and Plant Competition on Leucanthemum vulgare

To assess the impact of larval feeding by *D. aeratana* on Shasta daisy, and to assess the individual and combined impact of larval feeding by *D. aeratana* and plant competition on *L. vulgare*, we set up an impact experiment using potted plants. The Shasta daisy cultivar *L. × superbum* ‘Amelia’ was selected for this experiment because it had the highest attack rates by *D. aeratana* under no-choice and multiple-choice conditions (see Tables 1–3). Seeds from all three species were sown in December 2012. In February 2013, 68 *L. vulgare* seedlings were potted and to half of the pots one seedling of the grass *Poa pratensis* was added. The 32 *L.* × *superbum* plants were potted in April. All plants were potted in a mixture of garden soil (Selmaterra, Eric Schweizer AG, Switzerland), sand, and vermiculite (14:3:1) with 1 g/L of slow-release NPK fertiliser (Hauert Tardit 6M) added to the soil used for potting *L.* × *superbum*. In April, the 68 *L. vulgare* plants (34 with *P. pratensis* and 34 without) and the 32 *L. × superbum* plants were randomly allocated to two groups. Thirty *D. aeratana* larvae each were transferred to half of the *L. vulgare* plants grown with and without competition, as well as to half of the *L. × superbum* plants. No larvae were transferred to the remaining plants. At the same time, the number of rhizomes visible from above ground was recorded for each plant as a proxy of the initial plant size. After being kept in the laboratory for one day, the potted plants were embedded in sawdust in a garden bed laid out with a plastic sheet to prevent roots growing into the soil underneath. The garden bed was covered with a field cage (4 m × 2 m × 1.6 m) to protect the plants from other herbivores. In October 2013, when most of the stems had senesced, the flower heads of all *L. vulgare* and *L. × superbum* plants were counted and the length of the longest stem was measured. From April to May 2014, one year after setting up the experiment, all plants were individually covered with gauze bags and regularly checked for adult emergence. In July 2014, when no new flower heads were produced, the number of flower heads of each plant was counted, the length of the longest stem measured, and the above- and below-ground biomass were separately harvested, dried at 80 °C for 24 h, and then weighed.

### 2.5. Statistical Analyses

To examine whether larval survival (i.e., the proportion of transferred larvae that were recovered in autumn) under no-choice conditions differed between *L. vulgare* and *L. ircutianum* or between North American and European *L. vulgare* plants, we combined data from all ten years and analysed them using generalised linear mixed models (GLMMs) with a binomial error distribution and with plant population as a random factor. To investigate whether larval survival differed among populations of *L. vulgare* or *L. ircutianum*, we used generalised linear models (GLMs) with a quasi-binomial error distribution to account for overdispersion. Separate models were conducted for *L. vulgare* and *L. ircutianum* and likelihood ratio tests were used to compare the models with and without population as a fixed factor. Only data collected at CABI were used for these analyses.

To compare the proportion of eggs that developed to adults in the oviposition and development test between *L. vulgare*, *L. ircutianum*, and Shasta daisy, we used GLMs with binomial or quasi-binomial error distribution. To investigate whether the proportion of eggs that developed to adults differed among Shasta daisy cultivars, we used a likelihood ratio test to compare the model with and without the cultivar as a fixed factor.

To investigate whether the number of larvae (or eggs) found per plant differed between species or Shasta daisy cultivars in the multiple-choice cage and open-field tests, we conducted separate analyses for each of the tests (all cages set up in one year were considered as one test). To analyse the data from the multiple-choice cage tests, we used GLMMs with a negative binomial error distribution and cage as a random factor, and to analyse the data from the open-field tests we used quasi-Poisson GLMs. To investigate whether the number of larvae found per *L. vulgare* (in the open-field test set up in 2013) or Shasta daisy plant (in the open-field test set up in 2014) varied with the distance and geographic orientation from the release point, we used Poisson GLMs. To analyse whether the weight and instar of the larvae found on Shasta daisy differed from those found on *L. vulgare* rosettes, we combined the data for all Shasta daisy cultivars and conducted t-tests (for larval weight) or *χ*^2^-tests (to test for differences in the proportions of larvae found for each instar).

To investigate the impact of *D. aeratana* on the Shasta daisy cultivar *L. × superbum* ‘Amelia’ and the impact of *D. aeratana* and plant competition on *L. vulgare*, we used quasi-Poisson GLMs for the number of rhizomes and the number of flower heads and linear models with square-root transformed data for the above- and below-ground biomass and the length of the longest stem. Five *L. vulgare* and one *L.* × *superbum* plant that had been placed at one edge of the garden bed had on average a 12- (for *L. vulgare*) and 5- (for *L.* × *superbum*) times higher biomass than the other plants. The most likely explanation is that these plants were not located on the root-stopping mat that was placed in the garden bed and, therefore, had access to additional nutrients from the soil beneath. We therefore excluded these plants from the analyses. All statistical analyses were performed with the software R version 3.6.2 [36]. GLMMs were done using the function glmer in the lme4 package [37].

## 3. Results

### 3.1. No-Choice Larval Development Tests

Larval survival was similar on *L. vulgare* from North America and on *L. vulgare* from Europe (*z* = 0.9, df = 212, *P* = 0.4), however on *L. vulgare* slightly more of the larvae transferred in spring were recovered in autumn than on *L. ircutianum* (51.0 ± 1.9% vs. 45.2 ± 3.9%, *z* = 1.9, df = 380, *P* = 0.05). Larvae were found on all of the tested *L. vulgare* and *L. ircutianum* populations and the percentage of larvae recovered varied between 20% and 67% among populations (Table S1, *χ*^2^ = 31.0, df = 1, *P* = 0.8 for *L. vulgare* and *χ*^2^ = 18.1, df = 1, *P* = 0.1 for *L. ircutianum*).

Larvae of *D. aeratana* were also found on 11 of the 74 non-target species tested (Table 1). All of these plants belong to the tribe Anthemideae. Larvae were found on all of the tested Shasta daisy (*L.* × *superbum*) cultivars. Larval development varied among cultivars and was highest on the cultivar ‘Amelia’, where 30.7% of the transferred larvae were recovered in autumn, and the lowest on the cultivar ‘Becky’ where on average 2.5% of the transferred larvae were recovered (Table 1). A few larvae were also found on the North American native species *Matricaria discoidea* D.C. and *M. occidentalis* Greene, on the Australian native species *Cotula cotuloides* (Steetz) Druce, on the South African variety *C. vulgaris* var. *vulgaris*, which was tested in place of the Australian variety *australasica*, on the medicinal plants *Matricaria chamomilla* L., and *Tanacetum parthenium* (L.) Sch.-Bip., on the minor ornamental species *Ismelia carinata* (Schousboe) Sch.-Bip., *Leucanthemella serotina* (L.) Tzvelev, and *Mauranthemum paludosum* (Poir.) Vogt & Oberprieler, as well as on the introduced weed *Anthemis cotula* L. (Table 1). In addition, a single larva was found on a pile of soil when dissecting a plant of the minor ornamental species *Achillea ptarmica*. However, it is unclear whether this larva actually developed in this plant, or in a previously dissected plant. Since a total of 23 plants of this species were tested and no additional larvae were found on any of them, we consider this result an artefact.

Complete development to adult was observed on several Shasta daisy cultivars, on *A. cotula*, *L. serotina*, and on *T. parthenium*. All other non-target species that supported partial larval development senesced before development could be completed and only 3rd or 4th instar larvae and no 5th instar larvae were recovered. On *M. discoidea* larvae were only found in plants dissected in August, but not in plants dissected in September of the same year.

### 3.2. Oviposition and Development Test with Shasta Daisy in Field Cages

**Figure 2:**
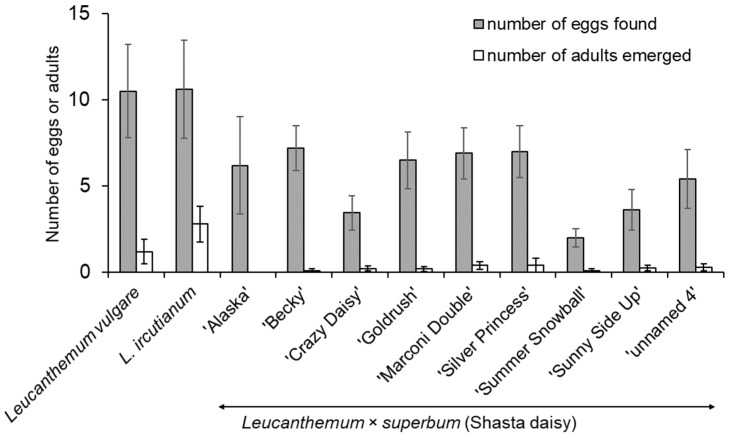
Mean (±SE) number of eggs found on and number of adults emerged from *Leucanthemum vulgare*, *L. ircutianum*, and nine Shasta daisy cultivars exposed to females of *Dichrorampha aeratana* in field cages.

In general, the results of the oviposition and development test with Shasta daisies were similar to those of the no-choice larval transfer tests, except that on average more eggs tended to develop to adults on *L. ircutianum* than on *L. vulgare* (*t* = 1.9, df = 18, *P* = 0.07, Figure 2). On average, fewer eggs developed to adults on Shasta daisy (all cultivars combined) than on *L. vulgare* and *L. ircutianum* (*z* = 3.0, df = 90, *P* = 0.003 and *z* = 6.8, df = 90, *P* < 0.001, respectively). Adult emergence was similar for all Shasta daisy cultivars (*χ*^2^ = 9.4, df = 1, *P* = 0.3).

### 3.3. Multiple-Choice Oviposition and Larval Development Tests in Field Cages

In the test conducted in 2012, similar numbers of eggs were found on *L. ircutianum* as on *L. vulgare* rosettes (*z* = 0.1, df = 31, *P* = 0.9), but approximately six and three times fewer eggs respectively were found on *L. × superbum* ‘unnamed 1’ and *L. × superbum* ‘Amelia’ than on *L. vulgare* rosettes (*z* = 3.4, df = 32, *P* < 0.001 and *z* = 2.4, df = 32, *P* = 0.01, Table 2). Similar numbers of larvae were found on *L. ircutianum* as on *L. vulgare* rosettes (*z* = 0.8, df = 32, *P* = 0.8), but approximately three times more larvae were found on *L. vulgare* with stems compared to rosettes (*z* = 3.9, df = 23, *P* < 0.001). Six to nineteen times fewer larvae were found on any of the three tested Shasta daisy cultivars compared to *L. vulgare* rosettes (all *P* < 0.001). Larvae found on Shasta daisies were on average 38% lighter than those found on *L. vulgare* (*t* = 4.2, df = 91, *P* < 0.001) and while more than 56.5% of the larvae found on *L. vulgare* were already in their fourth instar, 90.5% of the larvae found on the Shasta daisy cultivars were still in their third instar (*χ*^2^ = 12.5, df = 1, *P* < 0.001). In the remaining tests, larvae were found on 33–100% of the exposed *L. vulgare* plants, on 25% of the *M. paludosum* (creeping daisy) plants, and on single plants of *I. carinata*, *L. serotina*, and *M. occidentalis* (Table 2). No larvae were found on *A. ptarmica*, *A. cotula*, *C. cotuloides*, *M. chamomilla*, *M. discoidea*, and *T. parthenium* (Table 2).

### 3.4. Multiple-Choice Oviposition and Larval Development Tests under Open-Field Conditions

In the 2013 open-field test, where a large patch of Shasta daisies was surrounded by eight smaller patches of *L. vulgare* (Figure 1a), 26% more larvae were found on *L. vulgare* rosettes than on *L. vulgare* with stems (*t* = 2.3, df = 30, *P* = 0.03) and 3–35 times more larvae were found on *L. vulgare* rosettes than on any of the three Shasta daisy cultivars exposed (all *P* < 0.001, Table 3). Among the three Shasta daisy cultivars, significantly more larvae were found on the cultivar ‘Amelia’ than on the cultivars ‘Alaska’ and ‘unnamed 1’ (*t* = 2.7, df = 22, *P* = 0.01 and *t* = 3.5, df = 22, *P* = 0.002, respectively). Larvae found on Shasta daisies were of a similar weight as those found on *L. vulgare* rosettes (*t* = 1.6, df = 37, *P* = 0.1, Table 3) and the proportion of larvae found in their third, fourth, and fifth instar did not differ between *L. vulgare* rosettes and Shasta daisies (*χ*^2^ = 2.9, df = 2, *P* = 0.2, Table 3).

In the 2014 open-field test set up using patches of Shasta daisy and *L. vulgare* (Figure 1b), 11–73 times more larvae were found on *L. vulgare* rosettes than on any of the four Shasta daisy cultivars (all *P* < 0.001, Table 3). Larvae found on Shasta daisies were of a similar weight to those found on *L. vulgare* rosettes (*t* = 1.5, df = 16, *P* = 0.2, Table 3) and the proportion of larvae in their fourth and fifth instar did not differ between *L. vulgare* rosettes and Shasta daisies (*χ*^2^ = 0.04, df = 2, *P* = 0.8, Table 3).

In the open-field tests where individual plants of target and non-target species were randomly arranged (2014–2019, Figure 1c), six times more larvae were found on *L. vulgare* rosettes than on the simultaneously exposed *L. vulgare* with stems and nine times more than on *L. × superbum* ‘Silver Princess’ (*t* = 3.1, df = 16, *P* = 0.006 and *t* = 3.9, df = 15, *P* = 0.001, respectively) Larvae found on *L. × superbum* ‘Silver Princess’ were on average 35% lighter compared to those found on *L. vulgare* (*t* = 2.2, df = 14, *P =* 0.03, Table 3) and while all of the larvae found on *L. vulgare* rosettes were in their fourth instar, 75% of the larvae from *L. × superbum* ‘Silver Princess’ were still in their third instar (*χ*^2^ = 6.7, df = 1, *P* = 0.01, Table 3). Two larvae were also found on one *M. paludosum* plant, but no larvae were found on any of the other nine non-target species.

### 3.5. Impact of Dichrorampha aeratana on Shasta Daisy and Impact of D. aeratana and Plant Competition on Leucanthemum vulgare

**Figure 3:**
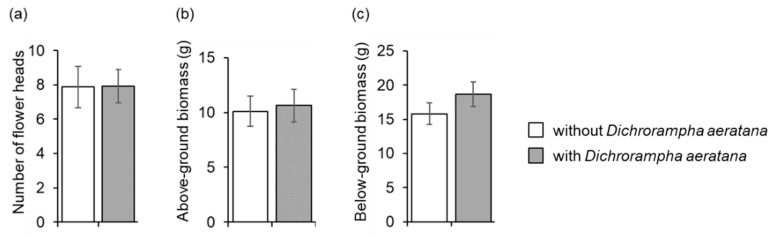
Mean (±SE) (**a**) number of flower heads, (**b**) above-ground biomass, and (**c**) below-ground biomass of the Shasta daisy cultivar *Leucanthemum* × *superbum* ‘Amelia’ infested with 30 larvae of *Dichrorampha aeratana* and non-infested plants. The plants were infested with larvae in April 2013 and measurements were taken in July 2014.

In April 2013, Shasta daisy plants exposed to *D. aeratana* larvae had a similar number of rhizomes as non-exposed plants (*t* = 0.2, df = 29, *P* = 0.8). In October, the number of flower heads and the length of the longest stem of Shasta daisy exposed to *D. aeratana* did not differ from those of non-exposed control plants (*t* = 0.4, df = 29, *P* = 0.7 and *t* = 0.2, df = 29, *P* = 0.8). Similarly, in July 2014, the number of flower heads, the length of the longest stem, and the above- and below-ground biomass did not differ between Shasta daisy plants exposed to *D. aeratana* and non-exposed control plants (*t* = 0.04, df = 29, *P* = 1.0, *t* = 0.04, df = 27, *P* = 1.0, *t* = 0.3, df = 29, *P* = 0.8 and *t* = 1.2, df = 29, *P* = 0.2 respectively, Figure 3). On average 1.0 ± 0.4 adults emerged per Shasta daisy plant in spring 2014.

**Figure 4:**
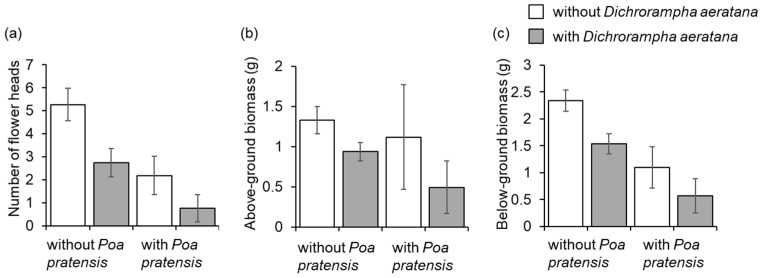
Mean (±SE) (**a**) number of flower heads, (**b**) above-ground biomass, and (**c**) below-ground biomass of *Leucanthemum vulgare* infested with 30 larvae of *Dichrorampha aeratana* and non-infested plants. Half of the infested and non-infested plants were grown in competition with *Poa pratensis.* The plants were infested with larvae in April 2013 and measurements were taken in July 2014.

In April 2013, the *L. vulgare* plants grown in competition with *P. pratensis* had on average 15% fewer rhizomes (*t* = 4.1, df = 60, *P* < 0.001) than those grown without competition but there were no differences in the number of rhizomes between plants exposed to *D. aeratana* larvae and non-exposed plants (*t* = 1.1, df = 60, *P* = 0.3). In October, *L. vulgare* plants grown in competition with *P. pratensis* had on average three times fewer flower heads and their longest stem was 26% shorter compared to *L. vulgare* plants grown without competition (*t* = 6.3, df = 60, *P* < 0.001 and *t* = 3.7, df = 59, *P* < 0.001, respectively), but none of these performance traits differed between plants exposed to *D. aeratana* and non-exposed control plants (*t* = 0.7, df = 60, *P* = 0.5 and *t* = 0.7, df = 59, *P* = 0.7, respectively). In July 2014, larval feeding by *D. aeratana* and competition with *P. pratensis* reduced the number of flower heads of *L. vulgare* by 54% and 64%, respectively (*t* = 2.3, df = 60, *P* = 0.02 and *t* = 3.0, df = 60, *P* = 0.004, respectively, Figure 4a), the above-ground biomass by 43% and 30% respectively (*t* = 1.9, df = 60, *P* = 0.06 and *t* = 3.2, df = 60, *P* = 0.002, Figure 4b) and the below ground biomass by 40% and 58%, respectively (*t* = 3.6, df = 60, *P* = 0.001 and *t* = 5.9, df = 60, *P* < 0.001, Figure 4c). Neither larval feeding by *D. aeratana* nor competition with *P. pratensis* had an influence on the length of the longest stem of *L. vulgare* (*t* = 0.7, df = 37, *P* = 0.5 and *t* = 0.8, df = 37, *P* = 0.4, respectively), both *P* > 0.1. The interaction between larval feeding by *D. aeratana* and competition from *P. pratensis* was not significant for any of the measured performance traits (all *P* > 0.1). In spring 2014, fewer adults emerged from *L. vulgare* grown in competition with *P. pratensis* than from those not grown in competition (0.8 ± 0.2 vs. 1.9 ± 0.4, *t* = 2.3, df = 30, *P* = 0.03).

## 4. Discussion

### 4.1. Host-Range of Dichrorampha aeratana

**Figure 5:**
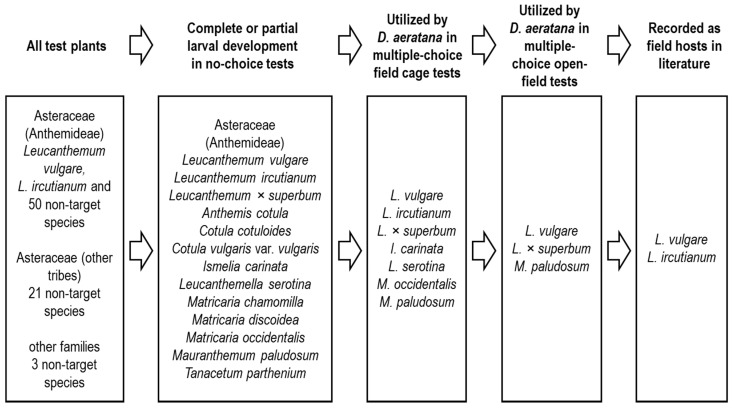
Summary of results of host-specificity tests with *Dichrorampha aeratana*. All plant species were first tested in no-choice larval development tests and all except one species (*Cotula vulgaris* var. *vulgaris*) that supported larval development in these tests were subsequently exposed to females in multiple-choice tests in field cages and under open-field conditions. *Leucanthemum ircutianum* was not tested under open-field condition.

From the 74 non-target species tested under no-choice conditions, partial or complete larval development was observed on 11 species from six genera, all within the tribe Anthemideae (Figure 5). Ten of these species were exposed under multiple-choice cage and open-field conditions and while five of them were accepted for oviposition in the cage tests, only two species (i.e., the ornamentals Shasta daisy and *M. paludosum*) were accepted for oviposition under open-field conditions (Figure 5). Under all test conditions, fewer larvae were found on non-target compared to *L. vulgare* plants. The two species with larvae found under open-field conditions both belong to the same subtribe as *L. vulgare*, which confirms that species closely related to the target weed have a higher risk of attack by potential biological control agents than more distantly related ones [38]. Our finding that the host-range of *D. aeratana* became progressively narrower from no-choice to multiple-choice cage to multiple-choice open-field conditions is in alignment with the concept that the host range of a specialist herbivore becomes further and further restricted, the more naturally it is able to display its host-selection behaviour [35,39]. A similar pattern was also found in studies investigating the potential of the sesiid moth *Negotinthia myrmosaeformis* (Herrich-Schaeffer) (syn.: *Tinthia myrmosaeformis*) as a biological control agent for *Potentilla recta* L. (Rosaceae) [40]. However, in this study, the reduction in the number of species and attack levels from no-choice to multiple-choice cage conditions was not as pronounced as in our tests with *D. aeratana*. In our tests, only one *D. aeratana* larva each was found on the three non-target species that were accepted as hosts when exposed in cages, but not in the open-field tests. This indicates that the large field cages used in our tests allowed for a close to natural oviposition behaviour by female moths.

All three designs used to investigate *D. aeratana*’s choice and use of Shasta daisy under open-field conditions gave comparable results, independent of the distance between exposed Shasta daisy or *L. vulgare* and the release point. This is in contrast to similar studies with other potential biological control agents. For example, in an open-field test with the root-feeding weevil *Cyphocleonus trisulcatus*, another potential biological control agent for *L. vulgare*, herbivory on *L. vulgare* and Shasta daisy by the weevil decreased with increasing distance (of up to 10 m) from the release point [15]. In addition, in a post-release open-field study with the root-feeding weevil *Mogulones crucifer* (Pallas), a biological control agent of *Cynoglossum officinale* L. (Boraginaceae), it was found that herbivory by the agent on both the target and a non-target species, *Hackelia micrantha* (Eastw.) J.L. Gentry declined with distance from the release points, with a greater decline for the non-target than for the target species [41]. In contrast to the findings of these studies, our results indicate that much larger distances (>10–20 m) would be necessary to evaluate whether the spatial distribution of *L. vulgare* and Shasta daisies influences the oviposition behaviour of the highly mobile *D. aeratana* females.

Under open-field conditions, significantly more larvae were found on the Shasta daisy cultivar ‘Amelia’ than on the other tested cultivars, and there was a tendency for a similar pattern under no-choice and multiple-choice cage conditions. However, the reason for this is unclear as there were no obvious morphological differences between Shasta daisy cultivars when in the rosette stage. Variation in the preference and/or performance among cultivars has been observed for several other biological control candidates. For example, the leaf beetle *Leptinotarsa texana* Schaeffer, a biological control agent for *Solanum elaeagnifolium* Cav. (Solanaceae), has been found to develop on only five of eight tested potato cultivars under no-choice conditions [42]. A similar pattern was also found for the beetles *Listronotus setosipennis* (Hustache) and *Zygogramma bicolorata* Pallister, two biological control agents for *Parthenium hysterophorus* L. (Asteraceae) that were found to oviposit on some, but not all, tested sunflower cultivars [43,44]. In contrast to these studies, we found no Shasta daisy cultivars that would be fully resistant to *D. aeratana*.

For most of the tested Shasta daisy cultivars, we found that they supported the complete development to adult, and for two of the females that emerged from Shasta daisy, we tested and confirmed that they would lay fertile eggs (Stutz, unpublished data). We can therefore not exclude that in the absence of *L. vulgare*, *D. aeratana* would be able to sustain a small population on Shasta daisy. However, since under open-field conditions, on average 3 to 90 times fewer larvae were found on Shasta daisy than on *L. vulgare*, the build-up of large populations on Shasta daisy is very unlikely. Complete development on the annual species *M. paludosum* is very unlikely, as this species usually senesces in August or September and no final instar larvae were found during host-specificity testing.

In the multiple-choice cage test and in one of the open-field tests, larvae found on Shasta daisies were on average lighter and occurred as earlier instars compared to those found on *L. vulgare*. The plants in both of these tests were dissected in August or early September. However, no differences in larval weight and instar were found between Shasta daisies and *L. vulgare* in the two open-field tests that were dissected in late September or October. It is unclear whether the smaller larvae found in August or early September would have been able to catch up on their development further into autumn or whether they would have died, but in general only very few dead larvae were found during our dissections.

Larvae of *D. aeratana* were able to develop on all tested *L. vulgare* and *L. ircutianum* populations from Europe and North America, which shows that although *D. aeratana* was initially collected on *L. ircutianum*, it is also well adapted to develop on *L. vulgare*. Interestingly, under open-field conditions, generally more larvae were found on *L. vulgare* rosettes than on *L. vulgare* with flowering stems, however, the opposite was the case under multiple-choice cage conditions (Table 2 and Table 3). These different results are most likely explained by the fact that plants with stems used in the open-field tests grew less vigorously than those exposed in the cage tests, and compared to plants in the rosette stage in general. The fact that *D. aeratana* accepts *L. vulgare* in the flowering and in the rosette stage for oviposition is an important prerequisite for the biological control of *L. vulgare* in North America, as *L. vulgare* is likely to occur in both phenostages during the oviposition period of *D. aeratana*.

### 4.2. Impact of Dichrorampha aeratana on the Non-Target Species Shasta Daisy

Our impact study showed that *D. aeratana* does not have an effect on the growth and flowering of the Shasta daisy cultivar ‘Amelia’, the cultivar with the highest attack rates, and we therefore conclude that it is also very unlikely that any of the other Shasta daisy cultivars would be negatively impacted by *D. aeratana*. As larval feeding occurred almost exclusively in the roots of Shasta daisies, there would also be no cosmetic damage. We therefore anticipate that *D. aeratana* will have no impact on the horticultural value of Shasta daisies in North America or Australia. Similar pre-release impact studies with non-target species have also been conducted with the psyllid *Arytinnis hakani* Loginova, a potential biological control agent for *Genista monspessulana* (L.) L.A.S.Johnson (Fabaceae) in California [45]. This species was found to develop and persist for at least two generations on several native *Lupinus* spp., while only affecting the growth and survival of the target weed *G. monspessulana* [45]. For the eriophyid mite *Aculus hyperici* (Liro), a biological control agent released against *Hypericum perforatum* L. (Hypericaceae) in Australia, post-release studies revealed that although this species regularly colonised the native non-target species *H. gramineum* G.Forst. in the field, and some minimal impact on the non-target species was observed under greenhouse conditions, no impact on the growth and reproduction of the non-target species was observed in the field [46,47,48].

### 4.3. Impact of Dichrorampha aeratana on the Target Species Leucanthemum vulgare

We found that both larval feeding by *D. aeratana* and competition from the grass *P. pratensis* significantly reduced the number of flower heads and the above- and below-ground biomass of *L. vulgare*. The largest impact on *L. vulgare* was observed when larval feeding by *D. aeratana* was combined with plant competition, suggesting that combining the use of competitive plants and biological control would be the most successful option to control *L. vulgare*. Several other studies found additive effects of biological control agents and plant competition [30]. For instance, combining the use of several biological control agents and the sowing of competitive plant species has been shown to be an effective method to control *Parthenium hysterophorus* in Australia [49,50].

Although it is difficult to extrapolate the impact of *D. aeratana* at the individual plant level to the population level, we expect that *D. aeratana* will contribute to the suppression of *L. vulgare* populations throughout its invaded range through a reduction in sexual reproduction, vegetative spread, and competitive ability. Only one other *Dichrorampha* species has been released as a biological control agent, *Dichrorampha odorata* Brown and Zachariades against *Chromolaena odorata* (L.) R.M.King and H.Rob. (Asteraceae) in South Africa. Laboratory studies with this shoot-boring species suggested a moderate reduction of plant height, flower production, and the competitiveness of *C. odorata* [51], but so far it has failed to establish in its introduced range, potentially due to poor climatic matching and/or due to high predation rates [52]. Univoltine root-feeding moths have, however, been successfully used in weed biological control, for example, *Pyropteron doryliformis* (Ochsenheimer 1808) (syn.: *Synansphecia doryliformis*) (Lepidoptera: Sesiidae) had a significant impact on *Rumex* species (Polygonaceae) populations in Australia [53]. A prerequisite for the successful control of an invasive weed is that the biological control agent reaches sufficiently high densities in the introduced range. Thus, the impact of *D. aeratana* on *L. vulgare* populations will ultimately depend to a large extent on its population dynamics in the area of introduction.

## 5. Conclusions

In conclusion, we consider *D. aeratana* to be a safe biological control agent for North America and Australia. The only non-target species that may be occasionally attacked by *D. aeratana* if released as a biological control agent are the two ornamental species Shasta daisy and *M. paludosum*. We expect that field attack rates on these two species will be very low and are therefore unlikely to affect their ornamental value. In addition, it is unlikely that any of the other, more distantly-related, economically important, or any native species will be attacked. We anticipate that *D. aeratana* would contribute to a meaningful reduction of *L. vulgare* vigour and reproductive output on both continents. We also expect that these effects will be enhanced if combined with other management options, such as the use of competitive plants.

## Figures and Tables

**Table 1:** Results of no-choice larval development tests with *Dichrorampha aeratana* conducted from 2011 to 2021. Plants were infested with five larvae in spring and either dissected for larvae in autumn or kept for adult emergence in the following spring. In addition, a subset of the larvae found on non-target and target plants were transferred onto new plants of the same plant species, which were then checked for adult emergence the following spring.

Plant Species		Plants Dissected for Larvae			Plants Kept for Adult Emergence or with Larvae Transferred Back on Same Plant Species		
		No. Plants	% Plants with Larvae	% Larvae Recovered/Plant (Mean ± SE)	No. Plants	% Plants with Adult Emergence	% Adults Emerged/Plant (Mean ± SE)
Family Asteraceae							
Tribe Anthemideae							
Subtribe Leucantheminae							
	*Leucanthemum vulgare* (Vaill.) Lam. (tests at CABI)	242	90.9	51.0 ± 1.9	59	81.4	36.4 ± 3.3
	*Leucanthemum vulgare* (tests at AAFC)	12	83.3	30.3 ± 0.6			
	*Leucanthemum vulgare* (tests at NSW DPI)	15	100.0	73.3 ± 4.7			
	*Leucanthemum ircutianum* (Turcz.) Turcz. ex DC.	141	85.8	44.5 ± 2.4	35	91.4	37.5 ± 4.2
	*Leucanthemum × superbum* (Bergmans ex J. Ingram) Kent ‘Alaska’	14	35.7	7.1 ± 2.7	10	10.0	2.0 ± 2.0
	*Leucanthemum × superbum* ‘Amelia’	15	73.3	30.7 ± 7.0	6	33.3	6.7 ± 4.2
	*Leucanthemum × superbum* ‘Becky’	8	12.5	2.5 ± 1.3			
	*Leucanthemum × superbum* ‘Crazy Daisy’	11	63.6	20.0 ± 7.6			
	*Leucanthemum × superbum* ‘Goldrush’	7	71.4	22.9 ± 8.1			
	*Leucanthemum × superbum* ‘Marconi Double’	5	20.0	4.0 ± 4.0	2	50.0	20.0 ± 20.0
	*Leucanthemum × superbum* ‘Silver Princess’	10	80.0	20.0 ± 5.2	3	0.0	
	*Leucanthemum × superbum* ‘Snow Lady’	4	25.5	5.0 ± 5.0	2	0.0	
	*Leucanthemum× superbum* ‘unnamed 1’ (from B & T Seeds)	10	50.0	14.4 ± 5.2	8	37.5	7.5 ± 3.7
	*Leucanthemum× superbum* ‘unnamed 2’ (from Eden Seeds)	10	70.0	17.5 ± 5.5			
	*Leucanthemum× superbum* ‘unnamed 3’ (from Australian Seed co.)	10	80.0	18.0 ± 3.6			
	*Mauranthemum paludosum* (Poir.) Vogt & Oberpr.	7	28.6	5.7 ± 3.7			
Subtribe Anthemidinae							
	*Anthemis arvensis* L.	11	0.0				
	*Anthemis cotula* L.	13	30.8	7.7 ± 5.1	10	10.0 ^d^	10.0 ± 10.0 ^d^
	*Anthemis tinctoria* L.	7	0.0				
	*Tanacetum camphoratum* Less. ^a^	7	0.0				
	*Tanacetum cinerariifolium* (Trevir.) Sch.Bip.	7	0.0				
	*Tanacetum huronense* Nutt. ^a^	7	0.0				
	*Tanacetum parthenium* (L.) Sch.Bip.	4	50.0	15.0 ± 9.6	5	20.0	4.0 ± 4.0
	*Tanacetum vulgare* L.	7	0.0				
	*Tripleurospermum inodorum* (L.) Sch.Bip.	7	0.0				
Subtribe Artemisiinae							
	*Arctanthemum arcticum* (L.) Tzvelev (ornamental)	7	0.0				
	*Arctanthemum arcticum* ^a^	9	0.0				
	*Artemisia absinthium* L.	9	0.0				
	*Artemisia arctica* Less. ^a^	2	0.0				
	*Artemisia biennis* Willd ^a^	7	0.0				
	*Artemisia californica* Less. ^a^	7	0.0				
	*Artemisia campestris* L. ^a^	7	0.0				
	*Artemisia cana* Pursh ^a^	7	0.0				
	*Artemisia dracunculus* L. ^a^	7	0.0				
	*Artemisia filifolia* Torr. ^a^	10	0.0				
	*Artemisia frigida* Willd. ^a^	7	0.0				
	*Artemisia ludoviciana* Nutt. ^a^	7	0.0				
	*Artemisia scopulorum* A. Gray ^a^	7	0.0				
	*Artemisia spinescens* D.C.Eaton ^a^	8	0.0				
	*Artemisia tridentata* Nutt. ^a^	7	0.0				
	*Artemisia vulgaris* L. ^a^	7	0.0				
	*Chrysanthemum × grandiflorum* (Ramat.) Kitam. ‘Garden Mums’	9	0.0				
	*Chrysanthemum × grandiflorum* ‘Morden Canary’	7	0.0				
	*Chrysanthemum × grandiflorum* ‘Morden Delight’	7	0.0				
	*Chrysanthemum × indicum* L.	5	0.0				
	*Hulteniella integrifolia* (Richardson) ^a^	12	0.0				
	*Leucanthemella serotina* (L.) Tzvelev	12	25.0	5.0 ± 2.6	4	25.0 ^e^	25.0 ± 25.0 ^e^
Subtribe Cotulinae							
	*Cotula alpina* (Hook.f.) Hook.f. ^b^	8	0.0				
	*Cotula australis* (Sieber ex Spreng.) Hook.f ^b^	17	0.0				
	*Cotula coronopifolia* L.	7	0.0				
	*Cotula cotuloides* (Steetz) Druce ^b^	8	16.7	3.3 ± 3.3			
	*Cotula vulgaris* Levyns var. *vulgaris* ^c^	11	9.1	1.8 ± 1.8			
	*Leptinella filicula* (Hook.f.) Hook.f ^b^	14	0.0				
	*Leptinella longipes* Hook.f. ^b^	12	0.0				
	*Leptinella reptans* D.G.Lloyd & C.J.Webb ^b^	7	0.0				
Subtribe Glebionidinae							
	*Argyranthemum frutescens* (L.) Sch.Bip.	13	0.0				
	*Glebionis coronaria* (L.) Cass. ex Spach	6	0.0				
	*Glebionis segetum* (L.) Fourr.	7	0.0				
	*Ismelia carinata* (Schousb.) Sch.Bip.	15	6.7	1.3 ± 1.3			
Subtribe Matricariinae							
	*Achillea alpina* L. ^a^	14	0.0				
	*Achillea borealis* Bong. ^a^	10	0.0				
	*Achillea ptarmica* L.	23	4.4 ^f^	0.9 ± 0.9 ^f^			
	*Matricaria chamomilla* L.	24	8.3	1.7 ± 1.2			
	*Matricaria discoidea* DC. ^a^	18	16.7	3.3 ± 1.8			
	*Matricaria occidentalis* Greene ^a^	15	13.3	2.6 ± 1.8	13	0.0	
Subtribe Santolininae							
	*Chamaemelum nobile* (L.) All.	7	0.0				
	*Santolina chamaecyparissus* L.	7	0.0				
Tribe Astereae							
	*Brachyscome aculeata* (Labill.) Less. ^b^	16	0.0				
	*Brachyscome multifida* DC. ^b^	5	0.0				
	*Calotis pubescens* (F.Muell. ex Benth.) N.G.Walsh & K.L. McDougall ^b^	12	0.0				
	*Erigeron compositus* Pursh ^a^	8	0.0				
	*Solidago nemoralis* Aiton ^a^	9	0.0				
	*Symphyotrichum laeve (L.) Á.Löve & D.Löve* ^a^	7	0.0				
Tribe Calenduleae							
	*Osteospermum ecklonis* (DC.) Norl.	5	0.0				
Tribe Cardueae							
	*Carthamus tinctorius* L.	7	0.0				
	*Cirsium flodmanii* (Rydb.) Arthur ^a^	6	0.0				
	*Cynara scolymus* L.	7	0.0				
Tribe Coreopsideae							
	*Coreopsis tinctoria* Nutt. ^a^	7	0.0				
Tribe Eupatorieae							
	*Eutrochium maculatum* (L.) E.E.Lamont ^a^	7	0.0				
Tribe Gnaphalieae							
	*Anaphalis margaritacea* (L.) Benth. ^a^	7	0.0				
Tribe Helenieae							
	*Helenium autumnale* L. ^a^	7	0.0				
Tribe Heliantheae							
	*Helianthus annuus* L. ^a^	14	0.0				
	*Echinacea purpurea* (L.) Moench	8	0.0				
Tribe Lactuceae							
	*Cichorium intybus* L.	7	0.0				
	*Lactuca sativa* L.	15	0.0				
Tribe Madieae							
	*Arnica chamissonis* Less. ^a^	9	0.0				
Tribe Senecioneae							
	*Senecio eremophilus* Richardson ^a^	7	0.0				
Tribe Tageteae							
	*Tagetes lucida* Cav.	7	0.0				
Family Apiaceae							
	*Daucus carota* L.	10	0.0				
	*Petroselinum crispum* (Mill.) Fuss	7	0.0				
Family Campanulaceae							
	*Lobelia cardinalis* L. ^a^	7	0.0				

^a^ Plant species native to North America. ^b^ Plant species native to Australia. ^c^ Plant species native to South Africa, tested in place of the Australian native *Cotula vulgaris* var. *australasica.* ^d^ It is unclear whether the one adult that emerged from *Anthemis cotula* completed its development on this plant species, or whether it emerged from a larva that was found on *A. cotula* in autumn and then transferred onto *Leucanthemum vulgare*. ^e^ Most likely an artefact. ^f^ The one adult emerged from a larva that was found on *Leucanthemella serotina* during dissections in autumn and transferred on a new *L. serotina* plant.

**Table 2:** Results of multiple-choice field cage tests conducted in 2012, 2013, 2016, 2017 and 2020. Six to twelve egg-laying females of *Dichrorampha aeratana* were released in each of the cages. Most *Leucanthemum vulgare* and *L. ircutianum* plants were in the rosette stage, while many of the non-target plants were already bolting or flowering.

	Plant Species/Cultivar	No. Plants	% Plants with Larvae	% Plants with Eggs or Larvae	No. Eggs/Plant (Mean ± SE)	No. Larvae/Plant (Mean ± SE)	No. Larvae with Weight and/or Instar Measured	Larval Weight (mg) (Mean ± SE)	% 3rd Instar	% 4th Instar
2012 (6 cages)										
*Leucanthemum vulgare* (rosettes)		18	77.8	94.4	8.2 ± 2.4	5.8 ± 1.3	69	2.1 ± 0.1	43.5	56.5
	*L. vulgare* (with stems)	9	100.0	100.0		16.9 ± 3.1	85	2.2 ± 0.1	43.5	56.5
	*L. ircutianum*	18	88.9	94.4	6.2 ± 1.3	6.9 ± 1.2	73	2.3 ± 0.1	43.8	56.2
	*L.* × *superbum* ‘Alaska’	18	33.3	50.0	5.8 ± 2.7	0.6 ± 0.2	8	1.3 ± 0.1	87.5	12.5
	*L.* × *superbum* ‘Amelia’	18	38.9	61.1	2.7 ± 0.7	0.9 ± 0.3	11	1.4 ± 0.1	90.9	9.1
	*L. × superbum* ‘unnamed 1’	18	20.0	33.3	1.3 ± 0.7	0.3 ± 0.1	2	1.0 ± 0.1	100.0	0.0
2013 (5 cages)										
	*L. vulgare*	15	100.0			10.3 ± 1.1	26	1.9 ± 0.2	73.1	26.9
	*L. ircutianum*	14	92.9			6.4 ± 1.5	18	2.2 ± 0.2	66.7	33.3
	*Anthemis cotula*	15	0.0			0.0				
	*Matricaria occidentalis* ^a^	15	6.7			0.07 ± 0.07	1	3.2	0.0	100.0
	*Leucanthemella serotina*	15	6.7			0.07 ± 0.07				
2016 (4 cages) ^b^										
	*L. vulgare*	6	33.3			2.7 ± 1.7	4	1.9 ± 0.1	25.0	75.0
	*Achillea ptarmica* ^c^	3	0.0			0.0				
	*Ismelia carinata*	6	16.7			0.2 ± 0.2	1	1.6	0.0	100.0
	*Matricaria chamomilla*	6	0.0			0.0				
	*Matricaria discoidea* ^a^	6	0.0			0.0				
2017 (4 cages)										
	*L. vulgare*	12	66.7			3.4 ± 1.1	9	2.2 ± 0.2	33.3	66.7
	*Ismelia carinata*	12	0.0			0.0				
	*Matricaria chamomilla*	12	0.0			0.0				
	*Matricaria discoidea* ^a^	12	0.0			0.0				
2020 (6 cages)										
	*L. vulgare*	18	94.4			5.8 ± 1.4	65		7.7 ^e^	78.5 ^e^
	*Cotula cotuloides* ^d^	14	0.0			0.0				
	*Mauranthemum paludosum*	16	25.0			0.4 ± 0.2	5		0.0	100
	*Tanacetum parthenium*	18	0.0			0.0				

^a^ Plant species native to North America. ^b^ Two cages had no larvae at all; data from these two cages are not presented. ^c^ Plant species included in tests even though we consider the test result of one larva found under no-choice condition as an artefact. ^d^ Plant species native to Australia. ^e^ 13.8% of the remaining larvae were in their 5th instar.

**Table 3:** Results of multiple-choice open-field tests conducted in 2013, 2014, 2017 and 2019. In April or May, 20 to 25 egg-laying females of *Dichrorampha aeratana* were released at the central point of each test. The plants were exposed for 2–4 weeks and dissected in autumn. Most *Leucanthemum* plants were in the rosette stage, while many of the other plants were already bolting or flowering.

Year	Plant Species/Cultivar	No. Plants	% Plants with Larvae	No. Larvae/Plant (Mean ± SE)	No. Larvae with Weight and/or Instar Measured	Larval Weight (mg) (Mean ± SE)	% 3rd Instar	% 4th Instar	% 5th Instar
2013 (design as in Figure 1a)									
	*Leucanthemum vulgare* (rosettes)	16	100.0	5.8 ± 0.7	26	5.4 ± 0.4	0.0	19.2	80.8
	*L. vulgare* (with stems)	16	93.8	3.8 ± 0.6	12	4.3 ± 0.4	8.3	58.3	33.3
	*L. × superbum* ‘Alaska’	12	8.3	0.2 ± 0.2	1	0.6	100.0	0.0	0.0
	*L.* × *superbum* ‘Amelia’	12	83.3	1.8 ± 0.4	11	4.8 ± 0.4	0.0	36.4	63.6
	*L.* × *superbum* ‘unnamed 1’	12	25.0	0.3 ± 0.1	1	3.5	0.0	0.0	100.0
2014 (design as in Figure 1b)									
	*L. vulgare* (rosettes)	15	100.0	9.1 ± 1.5	10	5.0 ± 0.6	0.0	40.0	60.0
	*L. vulgare* (with stems)	2	50.0	0.5 ± 0.5					
	*L. × superbum* ‘Alaska’	10	40.0	0.8 ± 0.4	6	4.0 ± 0.8	0.0	50.0	50.0
	*L. × superbum* ‘Becky’	10	20.0	0.2 ± 0.1	1	2.6	0.0	100.0	0.0
	*L.* × *superbum* ‘Crazy Daisy’	8	12.5	0.1 ± 0.1					
	*L.* × *superbum* ‘unnamed 4’	10	40.0	0.6 ± 0.3	1	4.2	0.0	0.0	100.0
2014^c^ (design as in Figure 1c)									
	*L. vulgare* (rosettes)	9	100.0	14.4 ± 2.4	12	3.1 ± 0.3	0.0	100.0	0.0
	*L. vulgare* (with stems)	7	71.4	2.3 ± 2.0					
	*L. × superbum* ‘Silver Princess’	8	50.0	1.6 ± 0.9	4	2.0 ± 0.2	75.0	25.0	0.0
	*Achillea ptarmica* ^a^	9	0.0	0.0					
	*Anthemis cotula*	7	0.0	0.0					
	*Matricaria occidentalis* ^b^	8	0.0	0.0					
	*Leucanthemella serotina*	9	0.0	0.0					
2017 (design as in Figure 1c)									
	*L. vulgare* (rosettes)	16	93.8	4.6 ± 0.8	12	2.5 ± 0.2	41.7	58.3	0.0
	*Ismelia carinata*	13	0	0.0					
	*Matricaria chamomilla*	16	0	0.0					
	*Matricaria discoidea* ^b^	16	0	0.0					
	*Matricaria occidentalis* ^b^	15	0	0.0					
2019 (design as in Figure 1c)									
	*L. vulgare* (rosettes)	15	80.0	2.9 ± 0.7	4		50.0	50.0	0.0
	*Cotula cotuloides* ^c^	8	0.0	0.0					
	*Mauranthemum paludosum*	16	6.3	0.1 ± 0.1	2		50.0	50.0	0.0
	*Tanacetum parthenium*	16	0.0	0.0					

^a^ Plant species included in tests even though we consider the test result of one larva found under no-choice condition as an artefact. ^b^ Plant species native to North America. ^c^ Plant species native to Australia.

## Data Availability

The data presented in this study are available on request from the corresponding author.

## Supplementary Materials

The following are available online at https://www.mdpi.com/article/10.3390/insects12050438/s1, File S1: Additional information on the methods. Table S1: Populations of *Leucanthemum vulgare* and *L. ircutianum* used as control plants for no-choice larval development tests conducted at CABI from 2011 to 2020.

## References

[1] Holm L., Pancho J.V., Herberger J.P., Plucknett D.L. (1979). A geographical Atlas of World Weeds.

[2] Clements D.R., Cole D.E., Darbyshire S., King J., McClay A. (2004). The biology of Canadian weeds. 128. *Leucanthemum vulgare* Lam. Can. J. Plant Sci..

[3] McDougall K., Wright G., Peach E. (2018). Coming to terms with Ox-eye Daisy (*Leucanthemum vulgare*) in Kosciuszko National Park, New South Wales. Ecol. Manag. Restor..

[4] Khuroo A.A., Malik A.H., Reshi Z.A., Dar G.H. (2010). From ornamental to detrimental: Plant invasion of *Leucanthemum vulgare* Lam. (Ox-eye Daisy) in Kashmir valley, India. Curr. Sci..

[5] Ahmad R., Khuroo A.A., Charles B., Hamid M., Rashid I., Aravind N. (2019). Global distribution modelling, invasion risk assessment and niche dynamics of *Leucanthemum vulgare* (Ox-eye Daisy) under climate change. Sci. Rep..

[6] Olson B.E., Wallander R.T., Sheley R.L., Petroff J.K. (1999). Oxeye daisy (*Chrysanthemum leucanthemum* L.). Biology and Management of Noxious Rangeland Weeds.

[7] McConnachie A.J., Peach E., Turner P.J., Stutz S., Schaffner U., Simmons A. (2015). The invasive weed ox-eye daisy, *Leucanthemum vulgare* Lam. (Asteraceae): Prospects for its management in New South Wales. Plant Prot. Q..

[8] McClay A.S., Stutz S., Schaffner U., Mason P., Gillespie D. (2013). *Leucanthemum vulgare* Lam., Oxeye Daisy (Asteraceae). Biological Control Programmes in Canada 2001–2012.

[9] Greuter W. Compositae (Pro Parte Majore). Euro+Med Plantbase: The Information Resource for Euro-Mediterranean Plant Diversity. http://ww2.bgbm.org/EuroPlusMed/.

[10] Fernald M.L. (1903). *Chrysanthemum leucanthemum* and the American white weed. Rhodora.

[11] Stutz S., Štajerová K., Hinz H.L., Müller-Schärer H., Schaffner U. (2016). Can enemy release explain the invasion success of the diploid *Leucanthemum vulgare* in North America?. Biol. Invasions.

[12] Mulligan G.A. (1958). Chromosome races in the *Chrysanthemum leucanthemum* complex. Rhodora.

[13] Thompson I.R. (2007). A taxonomic treatment of tribe Anthemideae (Asteraceae) in Australia. Muelleria.

[14] Oberprieler C., Himmelreich S., Källersjö M., Vallès J., Watson L.E., Vogt R., Funk V., Susanna A., Stuessy T., Bayer R. (2009). Anthemideae. Systematics, Evolution, and Biogeography of the Compositae.

[15] Stutz S., Hinz H.L., Schaffner U. (2020). Evaluation of *Cyphocleonus trisulcatus* (Coleoptera: Curculionidae) as a potential biological control agent for *Leucanthemum vulgare* in North America. J. Appl. Entomol..

[16] Tahara M. (1921). Cytologische Studien an einigen Kompositen. J. Coll. Sci. Imp. Univ. Tokyo Jpn..

[17] Hawke R.G. (2007). A Report on Leucanthemum × superbum and Related Daisies.

[18] Anderson N.O., Olsen R.T. (2015). A vast array of beauty: The accomplishments of the father of American ornamental breeding, Luther Burbank. HortScience.

[19] Bland K.P., Razowski J., Hancock E.F. (2015). The Moths and Butterflies of Great Britain and Ireland.

[20] Razowski J. (2003). Tortricidae (Lepidoptera) of Europe: Olethreutinae.

[21] Stutz S., Hinz H.L., Konowalik K., Müller-Schärer H., Oberprieler C., Schaffner U. (2016). Ploidy level in the genus *Leucanthemum* correlates with resistance to a specialist herbivore. Ecosphere.

[22] Sabourin M. (2009). First report of two palearctic *Dichrorampha* (Lepidoptera: Tortricidae) species for Vermont. VES News Newsl. Vt. Entomol. Soc..

[23] Hebert P.D., Ratnasingham S., Zakharov E.V., Telfer A.C., Levesque-Beaudin V., Milton M.A., Pedersen S., Jannetta P., deWaard J.R. (2016). Counting animal species with DNA barcodes: Canadian insects. Philos. Trans. R. Soc. B Biol. Sci..

[24] Schaffner U. (2001). Host range testing of insects for biological weed control: How can it be better interpreted?. Bioscience.

[25] Briese D.T. Open field host-specificity tests: Is “natural” good enough for risk assessment?. Proceedings of the Host specificity testing in Australasia: Towards Improved Assays for Biological Control, Paper from the Introduction of Exotic Biocontrol Agents-Recommendations on Host Specificity Testing Procedures in Australasia Workshop.

[26] Clement S.L., Cristofaro M. (1995). Open-field tests in host-specificity determination of insects for biological control of weeds. Biocontrol Sci. Technol..

[27] Schaffner U., Smith L., Cristofaro M. (2018). A review of open-field host range testing to evaluate non-target use by herbivorous biological control candidates. BioControl.

[28] Carvalheiro L.G., Buckley Y.M., Ventim R., Fowler S.V., Memmott J. (2008). Apparent competition can compromise the safety of highly specific biocontrol agents. Ecol. Lett..

[29] Pearson D.E., Callaway R.M. (2005). Indirect nontarget effects of host-specific biological control agents: Implications for biological control. Biol. Control.

[30] Sheppard A.W. The interaction between natural enemies and interspecific plant competition in the control of invasive pasture weeds. Proceedings of the IX International Symposium on Biological Control of Weeds.

[31] Cole D.E. (1998). Effect of Competition on Growth of ox-Eye Daisy (*Chrysanthemum leucanthemum* L.) in Pastures and Hay Land. Master’s. Thesis.

[32] Wapshere A.J. (1974). A strategy for evaluating the safety of organisms for biological weed control. Ann. Appl. Biol..

[33] Briese D.T., Spafford Jacob H., Briese D. (2003). The centrifugal phylogenetic method used to select plants for host-specificity testing of weed biological control agents: Can and should it be modernised?. Improving the Selection, Testing, and Evaluation of Weed Biological Control Agents.

[34] Kelch D.G., McClay A. Putting the phylogeny into the centrifugal phylogenetic method. Proceedings of the XI International Symposium on Biological Control of Weeds.

[35] Wapshere A.J. (1989). A testing sequence for reducing rejection of potential biological control agents for weeds. Ann. Appl. Biol..

[36] R Core Team (2019). R: A Language and Environment for Statistical Computing.

[37] Bates D., Mächler M., Bolker B., Walker S. (2015). Fitting linear mixed-effects models using lme4. J. Stat. softw..

[38] Pemberton R.W. (2000). Predictable risk to native plants in weed biological control. Oecologia.

[39] Marohasy J. (1998). The design and interpretation of host-specificity tests for weed biological control with particular reference to insect behaviour. Biocontrol News Inf..

[40] Turanli F., Schaffner U. (2004). Oviposition specificity of the specialist *Tinthia myrmosaeformis* under different degrees of behavioral restrictions. Biol. Control.

[41] Catton H.A., Lalonde R.G., De Clerck-Floate R.A. (2014). Differential host-finding abilities by a weed biocontrol insect create within-patch spatial refuges for nontarget plants. Environ. Entomol..

[42] Lefoe G., Haegi L., Rumpff L., Gopurenko D., Slater A.T., Butler K., Hauser C.E. (2020). Assessing the fundamental host-range of *Leptinotarsa texana* Schaeffer as an essential precursor to biological control risk analysis. Biol. Control.

[43] McConnachie A.J. (2015). Host range and risk assessment of *Zygogramma bicolorata*, a defoliating agent released in South Africa for the biological control of *Parthenium hysterophorus*. Biocontrol Sci. Technol..

[44] Wild C., McFadyen R., Tomley A., Willson B. (1992). The biology and host specificity of the stem-boring weevil *Listronotus setosipennis* [Col.: Curculionidae] A potential biocontrol agent for *Parthenium hysterophorus* [Asteraceae]. Entomophaga.

[45] Hogg B.N., Moran P.J., Smith L. (2019). Relative performance and impacts of the psyllid *Arytinnis hakani* (Hemiptera: Psyllidae) on nontarget plants and the target weed *Genista monspessulana* (Fabales: Fabaceae). Environ. Entomol..

[46] Willis A.J., Ash J.E., Groves R.H. (1993). Combined effects of two arthropod herbivores and water stress on growth of *Hypericum* species. Oecologia.

[47] Willis A., Ash J., Groves R. (1995). The effects of herbivory by a mite, *Aculus hyperici*, and nutrient deficiency on growth in *Hypericum* species. Aust. J. Bot..

[48] Willis A.J., Berentson P.R., Ash J.E. (2003). Impacts of a weed biocontrol agent on recovery from water stress in a target and a non-target *Hypericum* species. J. Appl. Ecol..

[49] Shabbir A., Dhileepan K., Zalucki M.P., O’Donnell C., Khan N., Hanif Z., Adkins S.W. (2015). The combined effect of biological control with plant competition on the management of parthenium weed (*Parthenium hysterophorus* L.). Pak. J. Bot..

[50] Shabbir A., Dhileepan K., O’Donnell C., Adkins S.W. (2013). Complementing biological control with plant suppression: Implications for improved management of parthenium weed (*Parthenium hysterophorus* L.). Biol. Control.

[51] Dube N., Uyi O., Zachariades C., Munyai T.C., Whitwell M. (2019). Impact of the shoot-boring moth *Dichrorampha odorata* (Lepidoptera: Tortricidae) on growth and reproductive potential of *Chromolaena odorata* (Asteraceae) in the laboratory. Biocontrol Sci. Technol..

[52] Zachariades C., Dube N., Nqayi S., Dlomo S., Uyi O. Attempts to establish *Dichrorampha odorata* on *Chromolaena odorata* in South Africa. Proceedings of the XV International Symposium on Biological Control of Weeds.

[53] Fogliani R., Strickland G. (2000). Biological Control of Dock: Enhanced Distribution of the Dock Moth.

