# Supplementary material for: Host Range and Impact of Dichrorampha aeratana, the First Potential Biological Control Agent for Leucanthemum vulgare in North America and Australia
Source: Insects. 2021 May 12;12(5):438. doi: 10.3390/insects12050438 (PMC8150849; doi:10.3390/insects12050438)
Supplement: Supplementary file 1 [file insects-12-00438-s001.zip › File S1.pdf]

Supplementary Materials File S1: Additional information on methods.

Insect rearing

For mating and oviposition of *Dichrorampha aeratana*, one or two freshly emerged females and one to three males were placed in transparent plastic cylinders (1.3 L) kept at room temperature. They were provided with a piece of wet cotton inserted in a vial of water and leaves of *Leucanthemum vulgare* or *L. ircutianum* inserted in moist florist sponge. Under these conditions, the females survived and laid eggs for approximately one week. The large majority of eggs were laid on the leaves, which were replaced at least three times per week. Since desiccation of the leaves would kill the eggs, the leaves were kept inserted in moist florist sponge until approximately eight days after oviposition and then transferred to tightly closed Petri dishes. At room temperature larvae emerged in approximately ten days.

Test plants

Most of the test plants and all control plants were grown from seeds. Non-target and target plants were usually sown two to four months before being used in tests. Some of the *L. vulgare* plants used in the multiple-choice cage tests and in the open-field tests were sown one year earlier to ensure that they produced flowering stems at the time the tests were conducted. The Shasta daisy cultivars 'Becky', 'Goldrush', 'Summer Snowball' and 'Sunny Side Up' as well as all three *Chrysanthemum* × *grandiflorum* cultivars were propagated from rhizomes or cuttings. All plants were grown in pots (diameter 10–14 cm, height 17 cm) in a mixture of garden soil, sand and vermiculite with 1 g/L of slow-release NPK fertilizer added for most species. Plants of *Hulteniella integrifolia* (Richardson) Tzvelev were collected in the field and grown in their original soil.

Host-specificity tests

Most of the no-choice tests and all the multiple-choice tests were conducted at CABI in Delémont, Switzerland between 2011 and 2020. Two to seven replicates of no-choice tests with *Argyranthemum frutescens* (L.) Sch.Bip., *Calotis pubescens* (F.Muell. ex Benth.) N.G.Walsh & K.L.McDougall, *Cotula vulgaris* Levyns var. *vulgaris, Chrysanthemum* × *indicum* L., *Brachyscome aculeata* (Labill.) Less., *B. multifida* DC., *Lactuca sativa* L., *Leptinella filicula* (Hook.f.) Hook.f., *Leptinella longipes* Hook.f., *Leptinella reptans* D.G.Lloyd & C.J.Webb *L.* × *superbum* 'unnamed 2'and 'unnamed 3' and *Osteospermum ecklonis* (DC.) Norl., were conducted in the quarantine at the New South Wales Department of Primary Industries in Orange, Australia from 2017–2021. All no-choice tests with *H. integrifolia* were conducted in the quarantine at Agriculture and Agri-Food Canada (AAFC) in Lethbridge, Canada in 2018.

Molecular analyses used to determine *Dichrorampha* larvae found in the open-field tests with *Dichrorampha aeratana*

The barcoding region of the mitochondrial cytochrome c oxidase subunit I gene (mtCOI) was used to confirm the identity of *D. aeratana* larvae and adults. Genomic DNA was extracted and amplified by Ivo Toševski (CABI) at the Institute for Plant Protection and Environment in Zemun, Serbia.
