# Supplementary material for: Host Range and Impact of Dichrorampha aeratana, the First Potential Biological Control Agent for Leucanthemum vulgare in North America and Australia
Source: Insects. 2021 May 12;12(5):438. doi: 10.3390/insects12050438 (PMC8150849; doi:10.3390/insects12050438)
Supplement: Supplementary file 1 [file insects-12-00438-s001.zip › Table S1.pdf]

Supplementary Materials Table S1: Populations of *Leucanthemum vulgare* and *L. ircutianum* used as control plants for no-choice larval development tests conducted at CABI from 2011 to 2020. All plants were infested with five larvae in spring and dissected for larvae in autumn.

Leucanthemum species	Origin	Population code	No. replicates	% larvae recovered / plant (mean ± SE)
	North America (Canada)			
L. vulgare	Alberta	Banff2	11	41.8 ± 5.0
L. vulgare	Alberta	Banff3	13	50.8 ± 11.5
L. vulgare	Alberta	Banff5	3	66.7 ± 17.6
L. vulgare	Alberta	TP 223	3	33.3 ± 17.6
L. vulgare	British Columbia	Coast #2	8	55.0 ± 9.8
L. vulgare	British Columbia	Lone Butte	9	62.2 ± 10.2
L. vulgare	British Columbia	CAN2	12	55.0 ± 9.3
L. vulgare	British Columbia	Wavey	24	45.8 ± 7.2
L. vulgare	Ontario	Moos	2	20.0 ± 0.0
	North America (USA)			
L. vulgare	Colorado	Douglas	16	62.5 ± 6.6
L. vulgare	Idaho	US16	4	45.0 ± 9.6
L. vulgare	Minnesota	SRLSP	4	40.0 ± 21.6
L. vulgare	Minnesota	USA5	12	51.7 ± 10.0
L. vulgare	Montana	Nine Pine	7	57.1 ± 11.1
L. vulgare	Montana	USA2	12	56.7 ± 7.3
L. vulgare	Oregon	USA1	12	53.3 ± 7.5
L. vulgare	Washington	US2	4	40.0 ± 11.5
L. vulgare	Washington	US6	2	60.0 ± 0.0
L. vulgare	Wyoming	Wiggins Fork	6	43.3 ± 3.3
	Australia			
L. vulgare	New South Wales	AUS	28	56.4 ± 5.1
	Europe			
L. vulgare	Czech Republic	CZ25	14	50.0 ± 10.8
L. vulgare	Germany	DE7	12	40.0 ± 7.0
L. vulgare	Poland	PL3VUL	12	61.7 ± 8.7
L. vulgare	Romania	RO5	12	40.0 ± 7.0
	North America (Canada)			
L. ircutianum	British Columbia	Barriere Lakes	15	56.0 ± 8.3
L. ircutianum	British Columbia	Telegr. Creek	21	41.0 ± 5.9
	Europe			
L. ircutianum	Switzerland	CH5	12	53.3 ± 7.9[a]
L. ircutianum	Czech Republic	CZ1	42	45.2 ± 3.9
L. ircutianum	Czech Republic	CZ29	15	33.3 ± 6.1
L. ircutianum	France	FR2	12	33.3 ± 8.3
L. ircutianum	Poland	PL3IRC	12	38.3 ± 10.0
L. ircutianum	Romania	RO7	12	60.0 ± 10.4
